# Active Neurodynamic Technique at Home in Patients with Knee Osteoarthritis: An Open Single Arm Clinical Trial

**DOI:** 10.3390/medicina60111857

**Published:** 2024-11-12

**Authors:** Beatriz Serrano-García, Carmen Belén Martínez-Cepa, Francisco Forriol, Juan Carlos Zuil-Escobar

**Affiliations:** 1 Escuela Internacional de Doctorado (CEINDO), Universidad San Pablo-CEU, CEU Universities, Urbanización Montepríncipe, 28660 Boadilla del Monte, Spain; 2 Departamento de Ciencias Médicas Básicas, Facultad de Medicina, Universidad San Pablo-CEU, CEU Universities, Urbanización Montepríncipe, 28660 Boadilla del Monte, Spain; 3 Departamento de Fisioterapia, Facultad de Medicina, Universidad San Pablo-CEU, CEU Universities, Urbanización Montepríncipe, 28660 Boadilla del Monte, Spain; jczuil@ceu.es; 4 Departamento de Ciencias Médicas Clínicas, Facultad de Medicina, Universidad San Pablo-CEU, CEU Universities, Urbanización Montepríncipe, 28660 Boadilla del Monte, Spain; fforriol@ceu.es

**Keywords:** physical therapy modalities, osteoarthritis, knee, pain, quality of life and self-care

## Abstract

*Background and Objectives*: Knee osteoarthritis (KO) stands as the third leading cause of disability among the elderly, causing pain, reduced quality of life, and decreased functionality. The objective of this study is to assess the effects of an active neurodynamic technique programme at home on pain, quality of life, and function among individuals with KO. *Materials and Methods*: Thirty-five participants (69.7% women) aged ≥50 years with KO (Kellgren–Lawrence grades I–II) performed a femoral nerve mobilization programme at home for 6–8 weeks (20 repetitions per day). Pain intensity, using the numerical rating scale (NRS), pressure pain thresholds (PPTs), central sensitization inventory (CSI), temporal assessment, pain modulation, Knee Injury and Osteoarthritis Outcome Score (KOOS), and the 12-item Short Form Survey questionnaire (SF-12) were collected before, after the intervention, and at one, three, six, and twelve months. *Results*: Participants improved significantly in pain (*p* < 0.05), with the improvement maintained throughout the follow-up in the NRS and for at least one month in the PPT. There were also statistically significant (*p* < 0.05) improvements in all subscales of the KOOS, which were maintained throughout the follow-up. Improvements were also found in the CSI and CPM. *Conclusions*: A home-based active neurodynamic programme for the femoral nerve has been demonstrated to yield positive effects on pain and function in patients with KO.

## 1. Introduction

Knee osteoarthritis (KO) is the third leading cause of disability in older people, causing pain, reduced quality of life, and reduced functionality [1]. Furthermore, the knee is the joint most commonly affected by osteoarthritis, accounting for 80% of cases [2]. KO is distinguished by the degeneration of articular cartilage, reduction in joint space, and the development of osteophytes [3]. Pain is a multifactorial phenomenon, involving neurophysiological, structural, and psychosocial factors [4]. Peripheral and central sensitization may play a role in KO [5,6,7]. Peripheral nociceptor sensitization is attributed to subchondral bone damage and synovial membrane inflammation, leading to an augmented perception of pain [8,9]. Conversely, central sensitization may result from the intense and sustained afferent input of nociception resulting from knee damage [10,11,12]. These mechanisms may be important components in the progression from acute to chronic pain [13].

At present, a definitive cure for KO remains elusive [1], leading to treatment strategies that primarily aim to reduce pain and improve function and quality of life [14,15]. The therapies to achieve these goals are primarily manual therapy and exercise [16]. This is due to the potential side effects associated with medications that have been included in practice guidelines over the past decade and the controversial efficacy of these medications in preserving cartilage and preventing disease progression [17].

Neurodynamic techniques (NTs) involve the reduction in nerve tension through the controlled sliding of nerves within the positions of various joint structures and mobilization [18]. Symptom reduction may result from increased sliding of the nerve and associated tissues, leading to increased nerve mobility and the mobilization of intraneural fluid [19]. Furthermore, Shacklock et al. [20] demonstrated the potential of NTs to enhance blood flow and nerve conduction. Previously literature has demonstrated that NTs can reduce pain and improve knee range of motion. Herrington et al. [21] found a statistically significant increase in knee range of motion through NTs in healthy women. On the other hand, Lau et al. [22] demonstrated the benefits of NTs on self-efficacy and pain relief in patients with rheumatoid arthritis. Despite the identified benefits of NTs, these techniques have not been previously studied in patients with KO.

Home-based programmes could play a crucial role in the management of patients with KO given the high prevalence and chronic nature of this disease. This approach would allow a greater number of patients to be reached, thereby reducing costs and time [23]. Previous studies have confirmed that home-based programmes, involving exercise, electrical stimulation, or health education, contribute to improved function and reduced symptoms in patients with KO [15,24,25]. These results are similar to those obtained with face-to-face counselling [25,26]. The effectiveness of home-based programmes depends on patient adherence and the feasibility of the intervention [27]. Research has shown that these programmes are more effective when delivered by physiotherapists [28].

No previous studies have studied the effects of a NT programme on pain, quality of life, and function in people suffering from KO. The aim of this study is to evaluate the effects of an NT programme on pain, quality of life, and function in people with KO. This investigation aims to examine the duration of the NT effect over time.

## 2. Materials and Methods

### 2.1. Design

The study design was an open single-arm clinical trial to evaluate the effects of a home-based NT programme in patients with KO (NCT05375448). The research received approval from the Ethics Committee of Fundación Jimenez Diaz Hospital (EO222-20_HRJB). Participants were informed of the methodology and objectives of the study, and each participant signed an informed consent form.

### 2.2. Participants

Inclusion criteria included patients aged 50 years or older, diagnosed with KO according to the American College of Rheumatology’s criteria [29], with knee pain, and grade I or II on the Kellgren–Lawrence radiographic scale [30]. Exclusion criteria included individuals suffering from chronic conditions considered to be perpetuating factors (e.g., fibromyalgia), those with conditions causing lower extremity pain, individuals who had taken analgesics within 24 h before evaluations, those who had undergone corticosteroid or local anesthetic infiltration in the year prior to the study or during the follow-up period, those using substances that could interfere with treatment, those with a previous diagnosis of neuropathy (lumbosacral plexus) or myopathy, those with contraindications to mobilization or exercise, and those with cognitive deficits (dementia, Alzheimer’s).

A sample size calculation was performed using G*Power 3.1, resulting in a sample size of 29; taking into account a 20% drop-out rate, the calculated sample size was 35 participants.

### 2.3. Intervention

**Figure 1:**
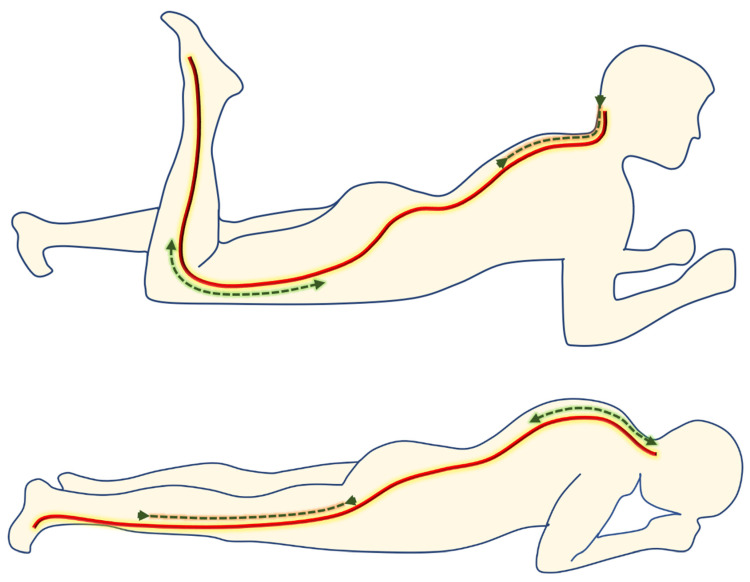
Mobilization of the femoral nerve. The arrows indicate the sliding of femoral nerve.

A physiotherapist guided the patients in performing active mobilization of the femoral nerve. The prescribed method for active neural mobilization involved assuming a prone position supported by the forearms with a slight extension of the spine, flexion of the knee, and extension of the cervical spine. This was followed by performing the opposite movements [31] (Figure 1). Participants were provided with a video illustrating the correct exercise technique and were instructed to perform the exercise for 6–8 weeks, with 20 repetitions per day (10 repetitions in the morning, 10 repetitions in the evening).

Treatment progress was monitored by self-report and weekly telephone follow-up. In cases where patients had questions or concerns, the physiotherapist conducted individual sessions to address these.

### 2.4. Variables

At baseline, demographic variables (age, sex, weight, height, body mass index (BMI), Kellgren–Lawrence radiographic scores, lower extremity deformity/dissymmetry, and analgesic use) were recorded. Outcome variables were assessed at baseline (T0), at the end of the treatment (T1), and at subsequent follow-up visits at 1 month (T2), 3 months (T3), 6 months (T4), and 12 months (T5). The intervals are T0–T1: 6–8 weeks; T1–T2: 1 month; T2–T3: 2 months; T3–T4: 3 months; and T4–T5: 6 months.

#### 2.4.1. Pain

The pain was assessed by the numerical rating scale (NRS), the pressure paint threshold (PPT), temporal summation (ST), and conditioned pain modulation (CPM). The Spanish central sensitization inventory (CSI) was also administrated to the participants.

The NRS, which has 11 intervals ranging from 0 (“no pain”) to 10 (“the worst pain imaginable”), has been shown to be a reliable tool in the elderly population and has a high correlation with other pain scales [32].

The PPT was measured using an analogue algometer (WAGNER, Greenwich, USA). The tip of the algometer (1 cm^2^/surface) was positioned perpendicular to the skin, and the pressure was increased at a rate of 1 kg/cm^2^/s until the first sensation of pain was reported. Three measurements were taken 30 s apart. Measurements were taken from three different locations: one point on the ipsilateral radial extensor of the carpus radials longus (5 cm from the lateral epicondyle) and two points on the patellar region (3 cm medial to the medial border and 3 cm lateral to the lateral border).

TS and CPM were assessed at the above points. For TS, 10 pulses of 1 s duration were applied to each of the three points, with a pressure increase of 2 kg/s at each pulse. Pain intensity was assessed using an NRS at the 1st, 5th, and 10th pulse. The TS was the difference, in percentage, between the 10th and the first [(TS 10th − TS 1st)/TS 1st] × 100. CPM was assessed after a five-minute rest period to allow for recovery between tests. Pressure was applied to the contralateral arm using a sphygmomanometer, which was gradually inflated at a rate of 20 mm Hg/s until the first perception of pain. The TS assessment was then repeated with the cuff pressure maintained on the arm, with a perceived pain intensity of 3/10 on the NRS. The CPM was the difference between the 10th score before occlusion and the 10th score during occlusion [33].

The CSI is a validated questionnaire designed to identify symptoms associated with central sensitization. It has two components: Scale A assesses 25 common symptoms in individuals with central sensitization, resulting in a score ranging from 0 to 100. This score was interpreted as follows: subclinical (0–29), mild (30–39), moderate (40–49), severe (50–59), and extreme (60–100). Scale B, which is not scored, collects information on previous diagnoses of specific diseases [33].

#### 2.4.2. Quality of Life and Function

The 12-item Short Form Survey (SF-12, Spanish version) questionnaire provides a comprehensive snapshot of the patient’s health status. It is characterized by its simplicity, quick completion time, and ease of scoring, providing information on capacity, well-being, and physical function in a practical, reliable, and valid way [34].

The Knee Injury and Osteoarthritis Outcome Score (KOOS, Spanish version) questionnaire has been validated and shown to be reliable in patients with KO. It consists of 42 items and includes five subscales: symptoms (KOOSS) with 7 items; pain (KOOSP) with 9 items; activities of daily living (KOOSADL) with 17 items; sport and recreational function (KOOSSR) with 5 items; and quality of life (KOOSQL) with 4 items. Each item has five response options: none, a little, moderate, severe, and extreme. The final score for the scale is derived by summing all the individual item scores. This cumulative score is then transformed into a scale ranging from 0 to 100, where 100 indicates no problems, and 0 indicates the most severe problems experienced by the patient [35].

The Mini-Cognitive Examination (MEC) is a screening questionnaire designed to assess cognitive impairment. It consists of 30 items divided into 11 sections and serves as the Spanish adaptation of the examination originally developed by Lobo et al. [36]. It was administered at baseline and at the end of the follow-up period due to the potential presence of dementia, which was an exclusion criterion. In addition, participants were asked to report any adverse effects of the intervention at follow-up.

### 2.5. Statistical Analysis

Statistical analysis was performed using the SPSS statistics (v. 29, IBM, SPSS Inc., Chicago, IL, USA). Statistical significance was set at *p* < 0.05. First, the Kolmogorov–Smirnov test was used to assess the normal distribution of quantitative variables. A descriptive study was performed, using means, standard deviations, medians, and interquartile ranges for quantitative variables and frequencies and percentages for qualitative data. Repeated measures ANOVA was used to compare variables at T0–T5 for variables showing a normal distribution, and the Friedman test was used in the opposite situation. A post hoc pairwise comparison was made using the Bonferroni test. Effect size was reported by η^2^_p_ in case of repeated measures ANOVA and Kendall’s W test value for the Friedman test.

## 3. Results

**Figure 2:**
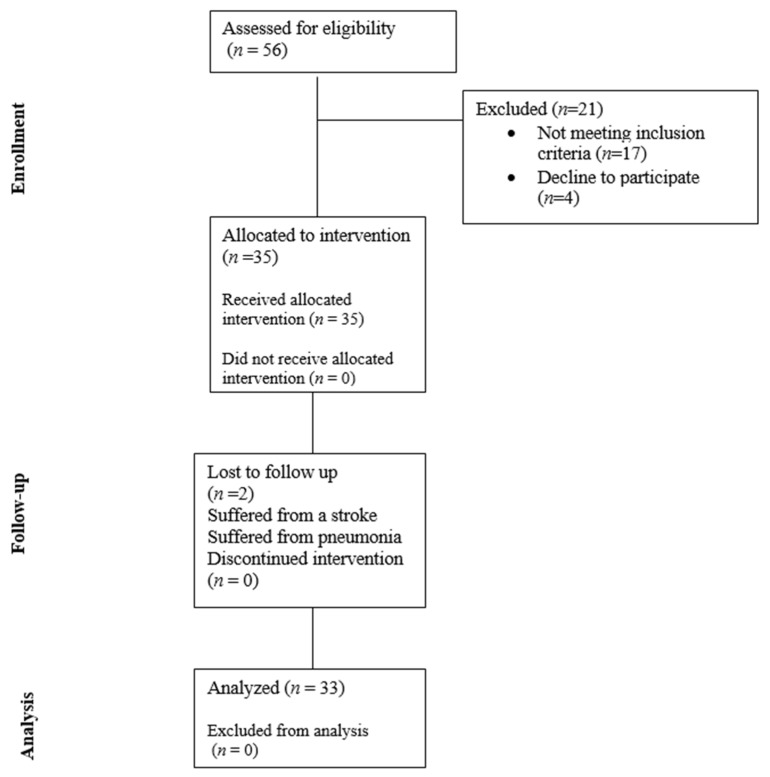
Flow chart of the participant enrollment, follow-up, and analysis.

Of a total of 56 potential participants, 35 participated in the study. Two patients were not assessed at all time points: one because of stroke and the other because of respiratory disease. Figure 2 shows the participant enrolment, follow-up, and analysis. The sample of 33 patients (23 women; 69.7%) showed a normal distribution of the demographic variables. Table 1 and Table S1 show the characteristics of the participants.

### 3.1. Pain

#### 3.1.1. NRS

**Figure 3:**
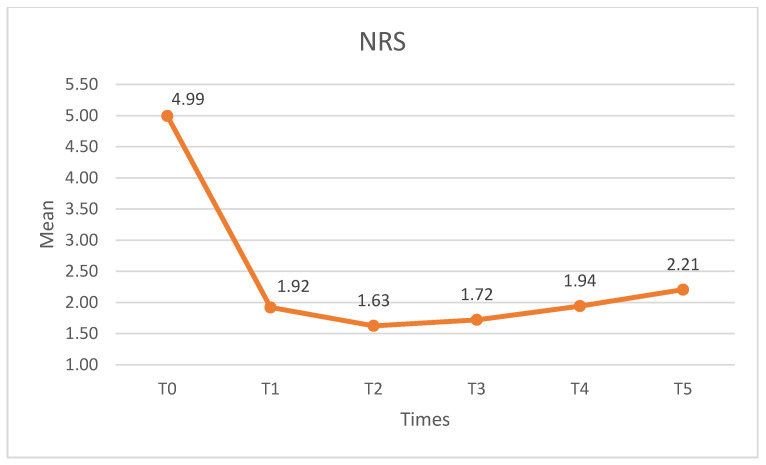
NRS at different time points. NRS: numerical rating scale.

Table 2 shows the means, standard deviations, medians, and interquartile ranges for the NRS and the PPT. The sample showed a normal distribution. Significant differences were observed in the NRS (F (2.292) = 55.282, *p* < 0.01, η^2^_p_ = 0.633, β-1 = 1). When comparing different time points, statistically significant differences (*p* < 0.05) were observed between the pre-intervention NRS and the post-intervention time points. However, no difference in the NRS was found in any of the post-intervention measurements. Figure 3 shows the NRS values at the different measurement times.

#### 3.1.2. PPT

##### Elbow PPT

**Figure 4:**
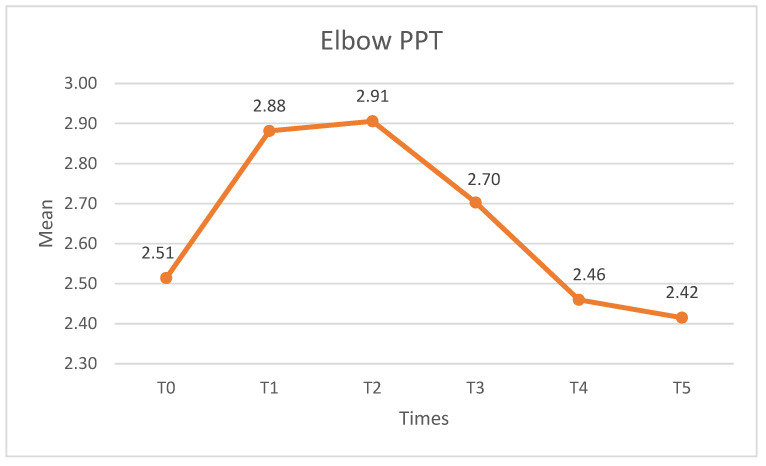
PPT elbow at different measurement times. PPT: pressure pain threshold.

Significant differences were observed in the PPT at the elbow (F (2.63) = 8525, *p* < 0.01, η^2^_p_ = 0.21, β-1 = 0.986). The pairwise comparison showed statistically significant differences (*p* < 0.05) between T0–T1 and T0–T2. The PPT scores increased statistically significantly after the intervention. Differences were also observed between T1–T4, T1–T5, T2–T4, T2–T5, and T3–T5 but, in this case, the PPT scores decreased. It therefore appears that the effects on the PPT at the elbow are only sustained during the first month after the intervention and begin to decrease after three months. Figure 4 shows the elbow PPT.

##### External Knee PPT

**Figure 5:**
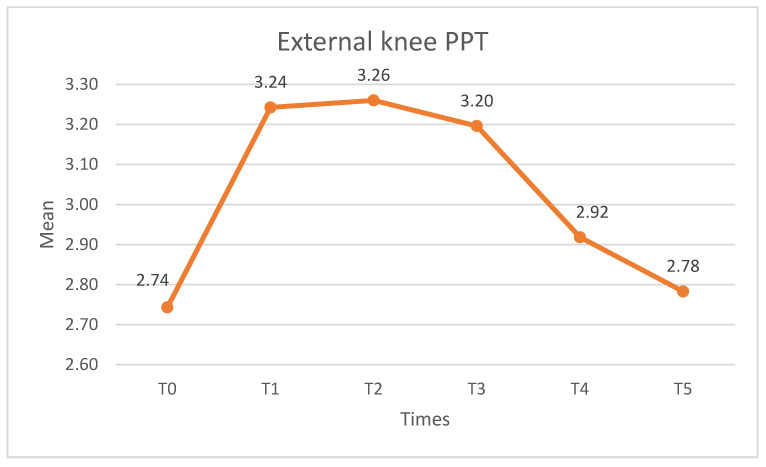
External knee PPT values. PPT: pressure pain threshold.

Significant differences were observed in the PPT at the lateral knee (X^2^ = 46.023, *p* < 0.01, W = 0.279). For pairwise comparisons, significant differences were found between T0–T1 (*p* < 0.01) and T0–T2 (*p* < 00.05) but not between T0 and the other times (*p* > 0.05). From T3, the PPT values start to decrease, with significant differences (*p* < 0.05) found in the pairs T1–T4, T1–T5, T2–T4, T2–T5, and T3–T5. Figure 5 shows the external knee PPT values.

##### Internal Knee PPT

**Figure 6:**
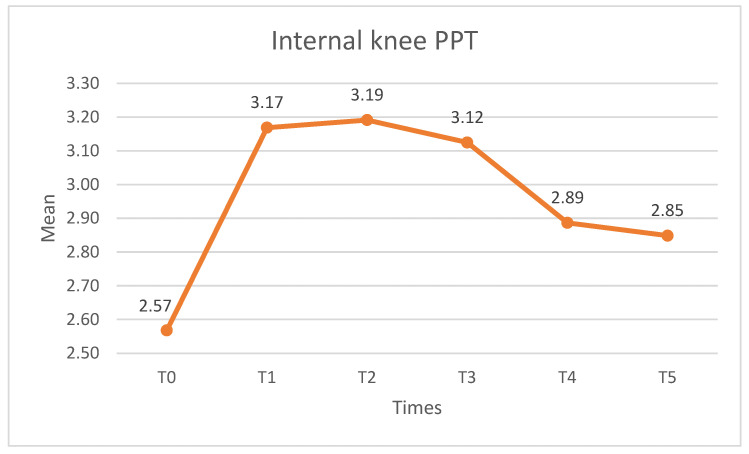
Internal knee ppt scores. PPT: pressure pain threshold.

Significant differences were observed in the PPT at the internal knee location (X^2^ = 35.898, *p* < 0.01, W = 0.218). The PPT values increased after the intervention, with significant differences found at T0–T1 (*p* < 0.01), T0–T2 (*p* < 0.05) and T0–T3 (*p* < 0.05); no statistically significant differences (*p* > 0.05) were found with respect to the pre-intervention measurement at either T4 or T5. The PPT decreased from T3 onwards, with statistically significant differences found between T1–T5, T2–T4, T2–T5, and T3–T5. Figure 6 shows the internal knee PPT.

#### 3.1.3. CSI

**Figure 7:**
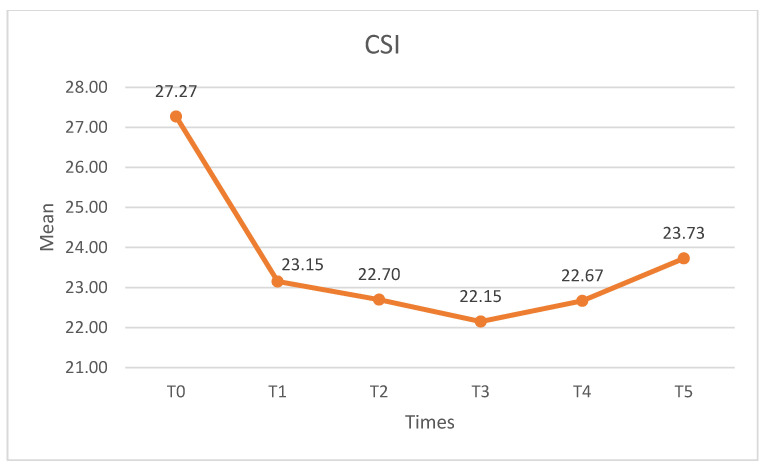
CSI scores. CSI: central sensitization inventory.

Table 3 shows the CSI (scale A) and ST values. Before the intervention, the participants showed subclinical values. Significant differences were observed (X^2^ = 38.805, *p* < 0.01, W = 0.235). In the pairwise comparison, statistically significant differences (*p* < 0.01) were found between T0 and all other measurement times except T5 (at 12 months follow-up). No differences were found in any of the post-intervention pairwise comparisons. The CSI (scale A) is shown in Figure 7.

Scale B of the CSI was also administered before the intervention. Seven participants (22.58%) had been diagnosed with any of the following conditions: temporomandibular disorder (6.1%), migraine or tension headaches (9.1%), irritable bowel syndrome (6.1%), neck injury (6.1%), anxiety or panic attacks (3.1%), and depression (6.1%). Three participants (9.1%) had one disorder, three (9.1%) had two, and only one participant (3.1%) had three. None of the 26 participants who did not have any disease before the intervention were diagnosed in the follow-up. Three of the participants who had a disorder before the intervention were diagnosed with a new disease at some point during the follow-up.

#### 3.1.4. ST

Before the intervention, no statistically significant differences (*p* > 0.05) in TS were found at the three sites. No significant differences (*p* > 0.05) were also found at any of the three locations during the follow-up. Table 3 shows mean, standard deviations, medians, and interquartile ranges.

#### 3.1.5. CPM

No statistically significant differences (*p* > 0.05) were found at the three locations in the CPM before the intervention. Statistically significant differences (X^2^ = 17.156, *p* < 0.05, W = 0.104) were found in the CPM located in the elbow, between T0 and T1. Regarding the CPM located in the external knee, although the Friedman test found significant differences (X^2^ = 12.133, *p* < 0.05, W = 0.074), the post hoc pairwise comparison did not find significant differences (*p* > 0.05). Finally, statistically significant differences were found in the CPM in the internal knee (X^2^ = 27.32, *p* < 0.01, W = 0.166) between T0–T1, T0–T2, T0–T3, T0–T4, and T0–T5. The Table 4 shows the CPM and SF-12 values.

### 3.2. Quality of Life and Function

#### 3.2.1. SF-12

**Figure 8:**
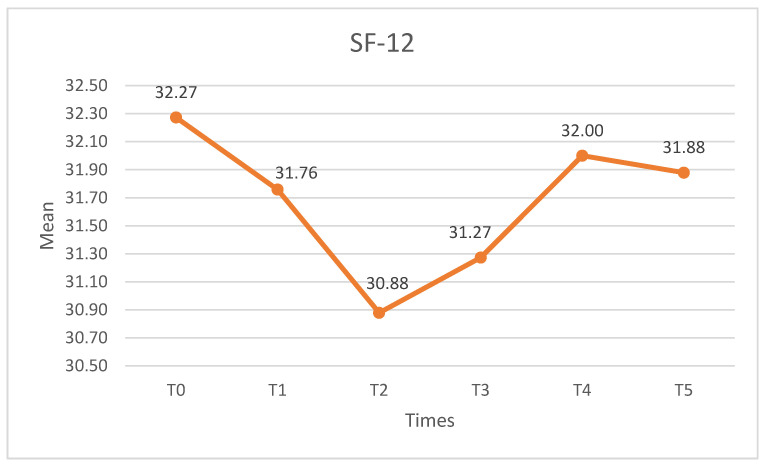
SF-12 scores. SF-12: version of the 12-item Short Form Survey.

Significant differences were observed in the SF-12 (X^2^ = 26.535, *p* < 0.01, W = 0.161). The SF-12 decreased after the intervention, with the lowest value found at the 1-month assessment (T2). Subsequently, scores increased again. Statistically significant differences (*p* < 0.05) were found between T2 and T0, and T4 and T5. Figure 8 shows the SF-12.

#### 3.2.2. KOOS

Table 5 shows the means, standard deviation, medians, and interquartile ranges for KOOS subscales.

##### KOOSS

**Figure 9:**
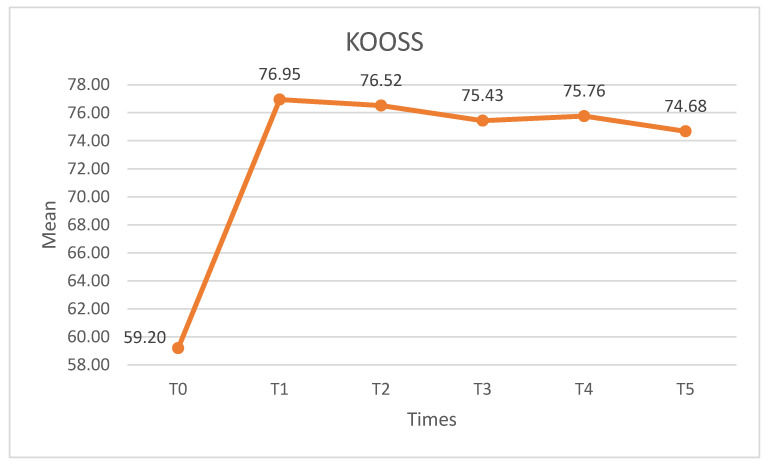
KOOSS scores. KOOSS: knee injury and osteoarthritis outcome score symptoms.

Significant differences were observed for the KOOSS (X^2^ = 56.911, *p* < 0.01, W = 0.345). In the pairwise comparisons, statistically significant differences (*p* < 0.05) were found between T0 and all post-intervention measures. The effect was maintained throughout the follow-up period, with no differences found in any of the post-intervention pairwise comparisons (Figure 9).

##### KOOSP

**Figure 10:**
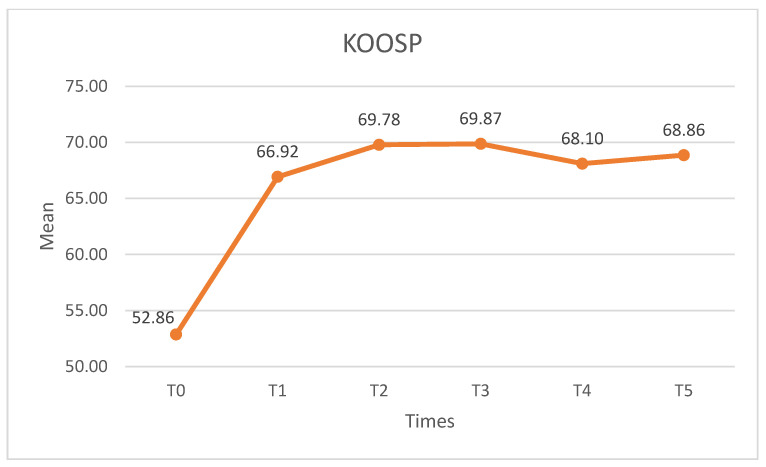
KOOSSPscores. KOOSSP: knee injury and osteoarthritis outcome score pain.

Significant differences were observed in KOOSP, with an effect size F (2.730) = 28.660, *p* < 0.01, η^2^_p_ = 0.472, β-1 = 1. The KOOSSR increased after the intervention (Figure 10), and statistically significant differences (*p* < 0.01) were found between T0 and the remaining time points. The effects on the KOOSSR were maintained throughout the follow-up period, and no statistically significant differences (*p* > 0.05) were found between the post-intervention pairwise comparisons.

##### KOOSADL

**Figure 11:**
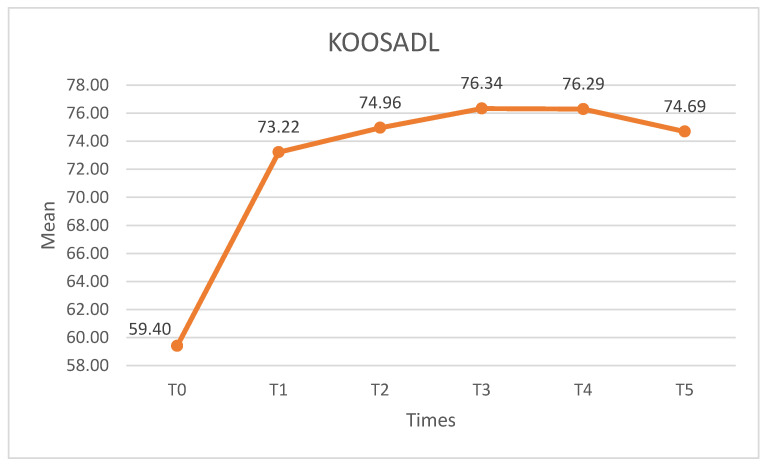
KOOSADL scores. KOOSADL: knee injury and osteoarthritis outcome score activities of daily living.

Significant differences were observed on the KOOSADL (X^2^ = 60.284, *p* < 0.01, W = 0.365) The score increased after the intervention, with statistically significant (*p* < 0.01) differences between T0 and the remaining time points. There were no statistically significant differences (*p* > 0.05) on any of the post-intervention measures (Figure 11).

##### KOOSSR

**Figure 12:**
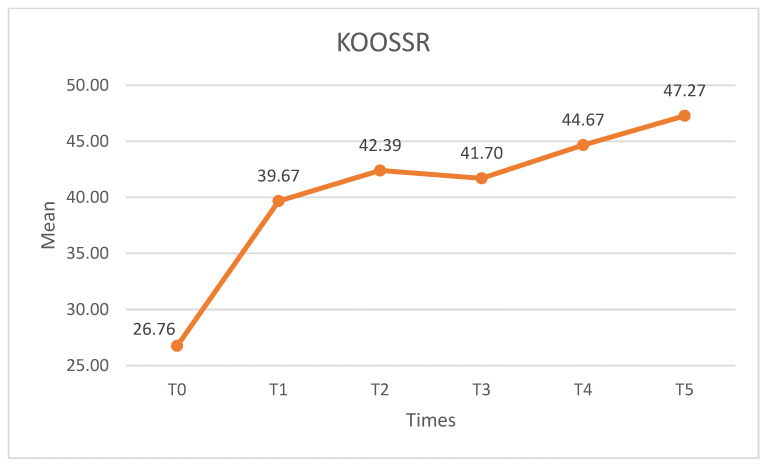
KOOSSR scores. KOOSSR: knee injury and osteoarthritis outcome score sports and recreational functioning.

As with the other subscales, statistically significant differences were observed for the KOOSSP (X^2^ = 38.779, *p* < 0.01, W = 0.235). The KOOSAD increased after the intervention, and statistically significant differences (*p* < 0.01) were found between T0 and the remaining measurement times. No statistically significant differences were found between any of the post-intervention pairs (*p* > 0.05) and the effect maintained throughout the follow-up (Figure 12).

##### KOOSQL

**Figure 13:**
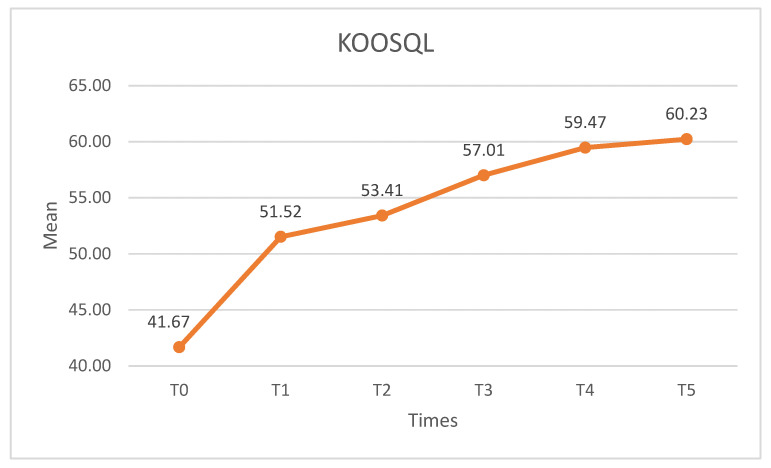
KOOSQL scores. KOOSQL: knee injury and osteoarthritis outcome score quality of life.

Finally, the KOOSQL subscale showed similar results (X^2^ = 50.050, *p* < 0.01, W = 0.303). After the intervention, the KOOSQL increased, with statistically significant differences (*p* < 0.01) found in pairwise comparisons between T0 and the remaining measurement times. There were no such differences (*p* > 0.05) when pairwise comparisons were made for the rest of the post-intervention and follow-up measures (Figure 13).

Tables S2–S4 show the mean differences in the pairwise comparison for all the variables evaluated. No adverse effects of the intervention were reported by any of the participants throughout the entire follow-up period.

## 4. Discussion

The current study investigated the effects of a home-based active NT. The active femoral NT showed positive effects on function and pain in patients with KO. No adverse effects of the home-based active NT were reported during patient follow-up.

Home-based rehabilitation offers patients the opportunity to adapt therapy to their daily routines, which is an important advantage, especially in the treatment of chronic degenerative diseases with prolonged treatment aimed at symptom management [37,38]. KO is one of the conditions that fall into this category and is associated with a significant economic burden for both healthcare systems and individuals. Home rehabilitation programmes play a key role in reducing these costs [39,40].

The advanced age of people with KO must be considered, as this may imply potential interactions between pharmacological treatments and other more aggressive therapies, as well as the increased risk of complications associated with joint replacement surgery [41,42].

The present study has innovative potential given the paucity of research related to the application of active NTs in patients with KO. Nevertheless, the existing literature has extensively demonstrated the beneficial effects of NTs in various pathologies. Lau et al. [22] showed how NTs on the knee nerves provided pain relief in individuals suffering from rheumatoid arthritis. On the other hand, Herriton et al. [21] showed how NTs helped to improve knee range of motion in healthy women. Furthermore, Sheereen et al. [43] highlighted that NTs were as effective as manual therapy in reducing pain in patients with carpal tunnel syndrome and surpassed it in improving nerve conduction velocity and hand function when exercise was incorporated into the interventions.

In our study, we assessed function, quality of life, and pain separately.

### 4.1. Pain

In terms of pain, we assessed self-perceived pain using the NRS and found a significant improvement after the intervention, which was maintained over time. To the best of our knowledge, this would be the first study to evaluate the use of an NT in KO using the NRS. The results obtained are consistent with the study by Arumugam et al. [44], in which NTs were used for pain relieve in individuals with lateral elbow pain, with statistically significant results after a single session of three mobilizations. These results highlight the interest in this technique for reducing self-perceived pain, as our study achieved statistically significant results with a 12-month follow-up. Our results are also consistent with previous studies using physiotherapy techniques in KO. For example, the study of Bhagat et al. [45] used manual therapy with Mulligan mobilizations and achieved statistically significant results after the intervention. Furthermore, studies using exercise as a therapy, such as that by Gür et al. [46], reported a significant reduction in pain after the intervention by using concentric and eccentric exercises. Similarly, Onwunzo et al. [47] found significant results after 6 weeks of quadriceps strengthening, and Praharsitha et al. [48] showed that hamstring strengthening also produced significant improvements after the intervention. Our results are also consistent with studies combining exercise with manual therapy, such as the study by Choudhary et al. [49], who found significant improvements following the use of exercise plus Maitland mobilizations. It is worth noting that while most of these studies evaluate results only in the short term, our study continues to evaluate the effects up to 12 months, allowing us to observe the long-term effectiveness of our intervention.

On the other hand, the following pain-related variables associated with possible sensitization in patients were measured: PPT, ST, CPM, and CSI. Regarding the PPT, this study showed statistically significant effects in both the knee and the elbow, with the effects on the elbow PPT being more sustained up to 3 months. To our knowledge, this is the first study to evaluate the PPT in people with KO using NTs. These results are consistent with those obtained in other body regions, as seen in the study by Kim et al. [50], who reported significant post-intervention PPT outcomes in patients with low back pain treated with NTs. These results are also consistent with those of Villafañe et al. [51], who found statistically significant improvements in patients with carpal tunnel syndrome treated with NTs, and with the study of Perdersini et al. [52], who found significant post-intervention improvements in patients with hand osteoarthritis treated with NTs. However, these effects did not last beyond 3 months, contrary to what was observed in our study. Our study is also consistent with other studies on KO that use different techniques. For example, in the study by Neelapala et al. [53], the use of isometric exercise produced statistically significant post-intervention results, with a 23% increase in the PPT. Tanaka et al. [54] also reported clinically and statistically significant results after massage intervention. Similarly, two studies by Pozsgai et al. [55,56], found significant post-intervention results using Maitland mobilizations. Additionally, Alkhawajah et al. [57] demonstrated statistically significant improvements, both locally and peripherally, with movement mobilization. Both our study and Alkhawajah et al. [57] confirmed the presence of hyperalgesia, both local and peripheral, with the latter lasting longer. The literature suggests that in addition to local physiological effects, joint mobilization activates descending inhibitory pathways from higher levels of the spinal cord, generating [56]. As with the previous measurements in our study, most studies in the literature typically assess results immediately after treatment, without analyzing the duration of the effect over time, similar to our study with a follow-up of up to 12 months. In this regard, Perdersini et al. measured results at 3 months but did not find significant long-term effects. Finally, according to the literature, normal PPT values in healthy subjects are 5.40 ± 1.49 kg/cm^2^ for the knee and 4.87 ± 1.37 kg/cm^2^ for the elbow [58], which are values well above those values obtained in our study: 2.57 ± 1.12 kg/cm^2^ for the knee and 2.51 ± 0.75 kg/cm^2^ for the elbow, suggesting possible pain sensitization in our patients.

However, the CSI scale A pre-intervention found that the scores of the participants were subclinical, indicating the absence of central sensitization. In addition, twenty-six participants (78.78%) did not have any diagnoses of diseases that could be related to central sensitization. The subclinical results in our patients may be related to the severity of symptoms, suggesting that central sensitization in individuals with KO may be related to the severity of the clinical presentation [7]. Therefore, it would be valuable for future studies to evaluate this variable in patients with more advanced KO or more severe symptoms to determine whether central sensitization plays a more significant role in the later stages of the disease.

In our study, statistically significant results were found in the CSI, which is consistent with the findings of Tirasci et al. [59], who observed improvements in the questionnaire in patients with KO through balance exercises, suggesting that the improvement in sensitization may be related to the analgesic effects of these exercises. This is also consistent with the study by Lluch et al. [33], who found improvements in the CSI in patients with KO through education and joint mobilization. As we noted before, the pre-treatment scores of our patients were subclinical, indicating the absence of central sensitization.

Regarding the ST, we did not find statistically significant differences in our study, between the three locations before the intervention, nor in the follow-up at any of the locations. These results are consistent with those reported in the study by Matesanz-García et al. [60], where no changes in the ST were observed after median nerve mobilization in healthy subjects. On the other hand, Bialosky et al. [61] found improvements in the ST through the use of NTs in patients with carpal tunnel syndrome, suggesting a favourable neurophysiological process. These results may be explained by the fact that our patients did not have severe degrees of the disease, showing normal responses in the ST in all the locations. The study by Fingleton et al. [7] suggests that central sensitization in individuals with KO may be related to the severity of the clinical presentation. It is possible that, in populations with more severe symptoms, central sensitization is more pronounced.

In terms of the CPM, statistically significant results were observed at the elbow before and after treatment, and at the medial knee when comparing pre-treatment measurements with those obtained after the intervention at 3, 6, and 12 months. These results are consistent with those reported by Courtney et al. [62], who demonstrated that joint mobilization could improve the CPM in individuals with KO. Other studies have also shown statistically significant improvements in the CPM with interventions such as surgery or the use of TENS [63,64,65]. For example, research by Vanegas et al. [66] suggested that deficits in the CPM may result from an imbalance between inhibitory and facilitatory signals in the nervous system, contributing to the perpetuation of pain. The results obtained support the hypothesis that NTs may play a crucial role in improving the CPM and, consequently, in pain modulation in patients with musculoskeletal disorders. Furthermore, the persistence of these results at 3, 6, and 12 months indicates that the benefits of these interventions are not only immediate but can also be sustained over time.

### 4.2. Quality of Life and Function

Regarding function and quality of life, based on the KOOS questionnaire, we observed improvements in all subscales up to 12 months. These findings are particularly relevant, as the KOOS includes five subscales, assessing pain, symptoms, activities of daily living, sport and recreational activity, and quality of life [35]. It is therefore a very useful tool to assess the effects of interventions and to follow-up on people suffering from KO. It should be noted that one of the subscales assesses pain, showing statistically significant improvements that are maintained up to one year after the intervention, which was also the case with the NRS. Therefore, the intervention has had positive effects on patient-reported pain.

This was the first study to evaluate the effects of an active NT programme on the KOOS in people suffering from KO. However, other authors have used this scale to assess various interventions in patients with KO. Islam et al. [67] found improvements in the KOOS questionnaire with manual therapy in patients with KO, with these improvements noted at the 20-day post-intervention assessment. Meanwhile, Lun et al. [68] observed similar improvements with therapeutic exercise after 3 months of intervention. It is important to emphasize that our study evaluates outcomes up to 12 months post-intervention, rather than immediately post-intervention as in the aforementioned studies, demonstrating the medium- and long-term effects of NTs in patients with KO.

In our study, no significant changes were observed in the SF-12 scores, which is a general health-related quality of life assessment tool that includes various physical and mental domains [69]. However, we found notable improvements in the subscale of the KOOS questionnaire, which evaluates the quality of life. The KOOS questionnaire measures different aspects of knee function, including pain, stiffness, physical function, and knee-related quality of life [35], having been designed to assess knee status in the context of injury and osteoarthritis. This specificity makes the KOOS particularly sensitive to changes in knee pathology and helps provide a detailed picture of how the condition affects patients’ daily lives. Our study suggests that the KOOS could be more effective than the SF-12 in capturing these changes.

### 4.3. Limitations

The main limitation of this study is that it is a single-arm clinical trial, so there is no control group and no randomization. Therefore, the results should be interpreted with caution. There is a need for a randomized controlled clinical trial that would not only confirm the results of this preliminary study but also allow a more precise assessment of the efficacy of the proposed intervention. However, it should be noted that single-arm clinical trials are useful when investigating new interventions, as in our case, and that they also overcome some ethical limitations of other designs, such as the fact that all participants receive the same intervention and have the right to choose [70]. Some of the limitations of single-arm clinical trials were controlled: patients did not receive any other intervention, and this is a degenerative disease where symptoms tend to worsen.

Combining and/or comparing NTs with other well-established therapeutic modalities, such as physical exercise or other types of manual therapy, whose benefits have been widely documented in the scientific literature, could also be considered in future research. A randomized clinical trial including these types of interventions, alongside and in front of NTs, would help to increase knowledge about the effects of different non-invasive techniques in the management of KO.

Another point to note is that our study included only people with grades I and II on the Kellgren–Lawrence radiographic scale. Our results, therefore, cannot be extended to people with grades III or IV on the Kellgren–Lawrence radiographic scale. A future study including people with these grades will be necessary.

## 5. Conclusions

An active home-based femoral nerve NT programme has been shown to have positive effects on pain and function in patients with KO. Of note, the improvement in subjective pain perception and function is maintained for 12 months. However, as the study did not include a control group, the results should be interpreted with caution. A further randomized controlled clinical trial should be conducted to verify these effects.

## Figures and Tables

**Table 1:** Demographic characteristics.

Demographic Characteristics (*n* = 33)		
	Mean	SD
Age	67.42	9.93
Height	1.65	0.09
Weight	70.29	14.43
BMI	25.50	3.87

SD: standard deviation.

**Table 2:** Means, standard deviations, medians, and interquartile ranges for NSR and PPT.

	NRS		Elbow PPT		External Knee PTT		Internal Knee PPT	
	Means (SD)	Medians (IQR)	Means (SD)	Medians (IQR)	Means (SD)	Medians (IQR)	Means (SD)	Medians (IQR)
**T0**	4.99 (1.84)	5 (3.45–6.05)	2.51 (0.75)	2.42 (1.94–3.04)	2.74 (1.16)	2.58 (1.79–3.5)	2.57 (1.12)	2.42 (1.58–3.33)
**T1**	1.92 (1.16)	1.75 (1–3)	2.88 (1.04)	2.67 (1.96–3.5)	3.24 (1.49)	2.75 (2.29–4.55)	3.17 (1.69)	2.5 (1.88–3.96)
**T2**	1.63 (1.12)	1.25 (0.55–2.68)	2.91 (1.10)	2.67 (2.08–3.37)	3.26 (1.66)	2.75 (2–3.68)	3.19 (1.78)	2.5 (1.79–3.84)
**T3**	1.72 (1.18)	1.5 (0.5–2.9)	2.70 (1.06)	2.75 (1.75–3.33)	2.5 (1.82–3.71)	2.92 (1.66)	3.12 (1.71)	2.58 (1.71–3.5)
**T4**	1.94 (1.11)	1.9 (1.2–3)	2.46 (0.94)	2.17 (1.75–3)	2.25 (1.87–1.67)	2.78 (1.58)	2.89 (1.60)	2.5 (1.84–3.42)
**T5**	2.21 (1.53)	2 (1–3.4)	2.42 (0.97)	2.17 (0.5–3.04)	2.78 (1.58)	2.33 (1.67–3.33)	2.85 (1.66)	2.5 (1.5–3.5)

IQR: interquartile range; NRS: numerical rating scale; PPT: pain pressure threshold; SD: standard deviation.

**Table 3:** Means, standard deviations, medians, and interquartile ranges for CSI and ST.

	CSI		Elbow TS		External Knee TS		Internal Knee TS	
	Means (SD)	Medians (IQR)	Means (SD)	Medians (IQR)	Means (SD)	Medians (IQR)	Means (SD)	Medians (IQR)
**T0**	27.27 (9.54)	28 (20–31.5)	92.78 (123.87)	66.67 (0–100)	94.13 (85.38)	66.67 (40–100)	96.55 (108.073)	60 (36.67–133.33)
**T1**	23.15 (9.33)	23 (15–28)	100.25 (84.66)	66.67 (55–116.67)	95.81 (70.95)	100 (50–133.33)	124.60 (117.73)	100 (66.67–150)
**T2**	22.70 (8.40)	21 (16.5–25)	91.67 (77.92)	100 (58.34–125)	89.9 (80.87)	100 (50–133.33)	79.07 (68.58)	75 (33.33–100)
**T3**	22.15 (7.50)	22 (15.5–25.5)	97.1 (155.85)	66.67 (43.75–100)	103.03 (75.57)	100 (50–129.17)	89.1 (89.78)	66.67 (50–100)
**T4**	22.67 (8.05)	22 (16–27.5)	87.02 (36.69)	100 (55–100)	105.91 (71.58)	100 (66.67–129.17)	91.51 (63.64)	100 (50–125)
**T5**	23.73 (8.11)	23 (18.5–26.5)	73.74 (50.31)	66.67 (33.33–100)	86.92 (9.13)	75 (41.67–100)	80.91 (58.54)	75 (50–100)

CSI: central sensitization inventory; IQR: interquartile range; SD: standard deviation; TS: temporal summation.

**Table 4:** Means, standard deviations, medians, and interquartile ranges for CPM and SF-12.

	Elbow CPM		External Knee CPM		Internal Knee CPM		SF-12	
	Means (SD)	Medians (IQR)	Means (SD)	Medians (IQR)	Means (SD)	Medians (IQR)	Means (SD)	Medians (IQR)
**T0**	−0.05 (2.31)	0 (−1–1)	0.68 (2.06)	0 (0–2)	0.09 (1.93)	0 (−1–1)	32.27 (2.31)	33 (30–34)
**T1**	1.55 (1.48)	1 (1–2.25)	1.39 (1.5)	1 (1–2)	1.15 (1.35)	1 (0–2)	31.76 (2.12)	31 (30.5–33)
**T2**	0.94 (1.5)	1 (0–2)	0.99 (1.41)	1 (0–2)	0.92 (1.19)	1 (0–2)	30.88 (1.98)	30 (30–32)
**T3**	0.53 (1.6)	1 (−5–2)	0.68 (1.42)	1 (0–1)	1.39 (1.64)	1 (0–2.5)	31.27 (1.86)	31 (30–33)
**T4**	0.98 (1.48)	1 (0–2)	1.35 (1.73)	1 (0–3)	1.66 (1.39)	2 (1–2)	32.00 (1.75)	32 (30.5–33.5)
**T5**	1.28 (1.22)	1 (0.7–2)	1.33 (1.61)	1 (0–2)	1.39 (1.3)	1 (1–2)	31.88 (1.62)	32 (31–33)

CPM: conditioned pain modulation; IQR: interquartile range; SD: standard deviation; SF-12: 12-item Short Form Survey.

**Table 5:** Means, standard deviations, medians, and interquartile ranges for KOOS subscales.

	KOOSS		KOOSP		KOOSADL		KOOSSR		KOOSQL	
	Means (SD)	Medians (IQR)	Means (SD)	Medians (IQR)	Means (SD)	Medians (IQR)	Means (SD)	Medians (IQR)	Means (SD)	Medians (IQR)
**T0**	59.20 (20.73)	60.71 (42.86–75)	52.86 (16.38)	50 (43.06–69.44)	59.40 (12.94)	60.29 (46.32–66.91)	26.76 (22.89)	25 (5–50)	41.67 (18.40)	37.5 (25–56.25)
**T1**	76.95 (14.18)	71.43 (67.86–91.07)	66.92 (13.33)	69.44 (54.17–75)	73.22 (15.10)	77.94 (58.82–83.82)	39.67 (24.38)	35 (15–60)	51.52 (18.49)	50 (37.5–62.5)
**T2**	76.52 (16.70)	75 (66.07–91.07)	69.78 (14.50)	69.44 (55.56–79.17)	74.96 (15.36)	77.94 (61.76–85.29)	42.40 (24.10)	35 (25–60)	53.40 (19.27)	56.25 (37.5–65.63)
**T3**	75.43 (16.65)	75 (62.5–91.07)	69.87 (13.86)	72.22 (55.56–80.56)	76.34 (14.97)	80.88 (63.97–86.76)	41.70 (22.59)	35 (21–55)	57 (18.34)	56.25 (43.75–68.75)
**T4**	75.76 (17.54)	75 (57.14–92.86)	68.10 (14.37)	66.67 (54.17–80.56)	76.29 (15.38)	83.82 (61.76–88.23)	44.67 (23.14)	40 (25–60)	59.47 (18.23)	62.5 (46.88–71.88)
**T5**	74.68 (17.89)	78.57 (57.1489.29)	68.86 (15.40)	66.67 (55.56–80.55)	74.69 (15.40)	73.52 (60.29–87.5)	47.27 (18.96)	40 (30–60)	60.23 (15.29)	62.5 (50–68.75)

IQR: interquartile range; KOOSADL: knee injury and osteoarthritis outcome activities of daily life; KOOSP: knee injury and osteoarthritis outcome pain; KOOSQL: knee injury and osteoarthritis outcome quality of life; KOOSS: knee injury and osteoarthritis outcome symptoms; KOOSSR: knee injury and osteoarthritis outcome sport and recreational function. SD: standard deviation.

## Data Availability

The data presented in this study are available on request from the corresponding author due to privacy.

## Supplementary Materials

The following supporting information can be downloaded at https://www.mdpi.com/article/10.3390/medicina60111857/s1, Table S1: Characteristics of the participants; Table S2: Mean differences in the pairwise comparison: numerical rating scale and pain pressure threshold at the locations; Table S3: Mean differences in pairwise comparison: temporal summation and conditioned pain modulation; Table S4: Mean differences in the pairwise comparison: Knee Injury and Osteoarthritis Outcome Score.
