# Supplementary material for: Active Neurodynamic Technique at Home in Patients with Knee Osteoarthritis: An Open Single Arm Clinical Trial
Source: Medicina (Kaunas). 2024 Nov 12;60(11):1857. doi: 10.3390/medicina60111857 (PMC11596390; doi:10.3390/medicina60111857)
Supplement: Supplementary file 1 [file medicina-60-01857-s001.zip › medicina-3283964-supplementary.pdf]

Table S1. Characteristics of the participants.

	**Frequency**	**Percentage**
**Studies**		
No education	1	3.0
Primary education	4	12.1
Secondary education	9	27.3
University	19	57.6
**Limb deformities**		
Yes	2	6.1
No	31	93.9
**Diagnostic**		
Radiography	25	75.8
Magnetic resonance	8	24.2
**Kellgren–Lawrence scale**		
I	10	30.3
II	23	69.7
**Treatment**		
Yes	4	12.1
No	29	87.9
**Analgesic treatment**		
Yes	4	12.1
Other treatment	29	87.9

Characteristics of the participants.

Table S2. Mean differences in the pairwise comparison: numerical rating scale and pain pressure threshold at the locations.

outcome	Time/time	T0	T1	T2	T3	T4	T5
NRS (mean difference)	T0		3.073	3.368	3.273	3.052	2.788
	T1	−3.073		0.295	0.200	−0.021	−0.285
	T2	−3.36 *	−0.295		−0.095	−0.317	−0.580
	T3	−3.273	−0.200	0.095		−0.221	−0.485
	T4	−3.052	0.021	0.317	0.221		−0.264
	T5	−2.788	0.285	0.580	0.485	0.264	
Elbow PPT (mean difference)	T0		−0.368	−0.393	−0.189	0.055	0.098
	T1	0.368		−0.025	0.178	0.422	0.466
	T2	0.393	0.025		0.204	0.447	0.491
	T3	0.189	−0.178	−0.204		0.244	0.288
	T4	−0.055	−0.422	−0.447	−0.244		0.044
	T5	−0.098	−0.466	−0.491	−0.288	−0.044	
External knee PPT (mean difference)	T0		−0.500	−0.518	−0.455	−0.177	−0.041
	T1	0.500		−0.018	0.045	0.324	0.460
	T2	0.518	0.018		0.063	0.341	0.477
	T3	0.455	−0.045	−0.063		0.278	0.415
	T4	0.177	−0.324	−0.341	−0.278		0.136
	T5	0.041	−0.460	−0.477	−0.415	−0.136	
Internal knee PPT (mean difference)	T0		−0.602	−0.624	−0.558	−0.318	−0.282
	T1	0.602		−0.022	0.044	0.283	0.319
	T2	0.624	0.022		0.065	0.305	0.341
	T3	0.558	−0.044	−0.065		0.240	0.276
	T4	0.318	−0.283	−0.305	−0.240		0.036
	T5	0.282	−0.319	−0.341	−0.276	−0.036	

NRS: numerical rating scale; PPT: pain pressure threshold.

Table S3. Mean differences in pairwise comparison: temporal summattion and conditioned pain modulation.

outcome	Time/time	T0	T1	T2	T3	T4	T5
CSI (mean difference)	T0		4.121	4.576	5.121	4.606	3.545
	T1	−4.121		0.455	1	0.485	−0.576
	T2	−4.576	−0.455		0.545	0.030	−1.030
	T3	−5.121	−1	−0.545		−0.515	−1.576
	T4	−4.606	−0.485	−0.030	0.515		−1.061
	T5	−3.545	0.576	1.030	1.576	1-061	
Elbow TS (mean difference)	T0		−7.475	1.110	−4.319	5.758	19.041
	T1	7.475		8.585	3.156	13.233	26.516
	T2	−1.110	−8.585		−5.429	4.647	17.930
	T3	4.319	−3.156	5.429		10.076	23.359
	T4	−5.758	−13.233	−4.647	−10.076		13.283
	T5	−19.041	−26.516	−17.930	−23.359	−13.283	
External knee TS (mean difference)	T0		−1.681	4.228	−8.903	−11.782	7.208
	T1	1-681		5.909	−7.223	−10.102	8.889
	T2	−4.228	−5.909		−13.132	−16.011	2.980
	T3	8.903	7.223	13.132		−2.879	16.112
	T4	11.782	10.102	16.011	2.879		18.991
	T5	−7.208	−8.889	−2.980	−16.112	−18.991	
Internal knee TS (mean difference)	T0		−28.045	17.482	7.461	5.036	15.642
	T1	28.045		45.527	35.505	33.081	43.687
	T2	−17.482	−45.527		−10.022	−12.446	−1.840
	T3	−7.461	−35.505	10.022		−2.424	8.182
	T4	−5.036	−33.081	12.446	2.424		10.606
	T5	−15.642	−43.687	1.840	−8.182	−10.606	
Elbow CPM (mean difference)	T0		−1.591	−0.985	−0.576	−1.030	−1.330
	T1	1.591		0.606	1.015	0.561	0.261
	T2	0.985	0.606		0.409	−0.045	−0.345
	T3	0.576	−1.015	-0.409		−0.455	−0.755
	T4	1.030	−0.561	0.045	0.455		−0.300
	T5	1.330	−0.261	0.345	0.755	0.300	
External knee CPM (mean difference)	T0		−0.712	−0.312	0	−0.664	−0.652
	T1	0.712		0.400	0.712	0.048	0.061
	T2	0.312	−0.400		0.312	−0.352	−0.339
	T3	0	−0.712	−0.312		−0.664	−0.652
	T4	0.664	−0.048	0.352	0.664		0.012
	T5	0.652	−0.061	0.339	0.652	−0.012	
Internal knee CPM (mean difference)	T0		−1.061	−0.833	−1.303	−1.573	−1.300
	T1	1.061		0.227	−0.242	−0.512	−0.239
	T2	0.833	−0.227		−0.470	−0.739	−0.467
	T3	1.303	0.242	0.470		−0.270	0.03
	T4	1.573	0.512	0.739	0.270		0.273
	T5	1.300	0.239	0.467	−0.03	−0.273	

CPM: conditioned pain modulation; TS: temporal summation.

Table S4. Mean differences in the pairwise comparison: Knee Injury and Osteoarthritis Outcome Score.

outcome	Time/time	T0	T1	T2	T3	T4	T5
SF-12 (mean difference)	T0		0.515	1.394	1	0.273	0.394
	T1	−0.515		0.879	0.485	−0.242	−0.121
	T2	−1.394	−0.879		−0.394	−1.121	-1
	T3	-1	−0.485	0.394		−0.727	−0.606
	T4	−0.273	0.242	1.121	0.727		0.121
	T5	−0.394	0.121	1	0.606	−0.121	
KOOSS (mean difference)	T0		−17.749	−17.316	−16.234	−16.558	−15.476
	T1	17.749		0.433	1.515	1.190	2.273
	T2	17.316	−0.433		1.082	0.758	1.840
	T3	16.234	−1.515	−1.082		−0.325	0.758
	T4	16.558	−1.190	−0.758	0.325		1.082
	T5	15.476	−2-273	−1.840	−0.758	−1.082	
KOOSR (mean difference)	T0		−14.057	−16.919	−17.003	−15.236	−15.993
	T1	14.057		−2.862	−2.946	−1.178	−1.936
	T2	16.919	2.862		−0.084	1.684	0.926
	T3	17.003	2.946	0.084		1.768	1.010
	T4	15.236	1.178	−1.684	−1.768		−0.758
	T5	15.993	1.936	−0.926	−1.010	0.758	
KOOSADL (mean difference)	T0		−3.815	−15.553	−16.934	−16.889	−15.285
	T1	13.815		−1.738	−3.119	−3.075	−1.471
	T2	−15.553	−1.738		−1.381	−1.337	0.267
	T3	−16.934	−3.119	1.381		0.045	1.649
	T4	16.889	3.075	−1.337	-0.045		1.604
	T5	15.285	1.471	0.267	1.649	1.604	
KOOSSP (mean difference)	T0		−12.909	−15.636	−14.939	−17.909	−20.515
	T1	12.909		−2.727	−2.030	-5	−7.606
	T2	15.636	2.727		0.697	−2.273	−4.879
	T3	14.939	2.030	-0.697		−2.970	−5.576
	T4	17.909	5	2.273	2.970		−2.606
	T5	20.515	7.606	4.879	5.576	2.606	
KOOSQL (mean difference)	T0		−9.84 (−18.0:−1.62)	−11.742 (	−15.341	−17.083	−18.561
	T1	9.848		−1.894	−5.492	−7.955	−8.712
	T2	11.742	1.894		−3.598	−6.061	−6.818
	T3	15.341	5.492	3.598		−2.462	−3.220
	T4	17.083	7.955	6.061	2.462		−0.758
	T5	18.561	8.712	6.818	3.220	0.758	

KOOSALD: knee injury and osteoarthritis outcome activities of daily life; KOOSP: knee injury and osteoarthritis outcome pain; KOOSQL: knee injury and osteoarthritis outcome quality of life; KOOSS: knee injury and osteoarthritis outcome symptoms; KOOSSR: knee injury and osteoarthritis outcome sport and recreational function.
